# Continuing medical education in epileptology: The Level 1‐2‐3 experience of the ILAE academy

**DOI:** 10.1002/epd2.70045

**Published:** 2025-05-27

**Authors:** Ingmar Blümcke, Eva Biesel, Samuel Wiebe, Sandor Beniczky, Jo M. Wilmshurst, Man Mohan Mehndiratta, Ali A. Asadi‐Pooya, Christian Brandt, Alexis Arzimanoglou, Gagandeep Singh, the ILAE Academy content development faculty, Raidah Al Baradie, Carmen Barba, Jocelyn F. Bautista, Sallie Baxendale, Elena Belousova, Isabella Brambilla, Jorge G. Burneo, Leonor Cabral‐Lim, Jaime Carrizosa, Jaume Campistol, Hannah Cock, Marion Comajuan, Helen Cross, Emilija Cvetkovska, Valentina De Giorgis, Luca De Palma, Kirsten A. Donald, Georg Dorfmüller, Guadalupe Fernandez Baca Vaca, Jessica Fesler, Irene García Morales, Christina Giavasi, Maria Gogou, Patricia Gómez Iglesias, Samson Gwer, Till Hartlieb, Martin Holtkamp, Floor E. Jansen, Bosanka Jocic‐Jakubi, Philippe Kahane, Ioannis Karakis, Takafumi Kubota, Joshua Laing, Tatjana Liakina, Aileen McGonigal, Rosa Michaelis, Ioana Mindruta, Andrew Neal, Maureen Njoroge, Irina Oane, Terence O’Brien, Finbar O’Callaghan, Manuela Ochoa Urrea, Nicholas Odero, Sebastián Ortiz, Karine Ostrowsky‐Coste, Maria T. Papadopoulou, Tiziana Pisano, Anna Rada, Stefan Rampp, Markus Reuber, Sylvain Rheims, Andrea O. Rossetti, Guido Rubboli, Victoria San Antonio‐Arce, Jan‐Christoph Schoene‐Bake, Artem Sharkov, Hugh D. Simpson, Mary Lou Smith, Ana Suller Marti, Rainer Surges, Roland Thijs, Joseph Toulouse, Bart van den Munckhof, Bernd J. Vorderwülbecke, Alistair Wardrope, Peter Wolf, Elza Márcia Yacubian

**Affiliations:** ^1^ Member of the ERN EpiCARE, Institute of Neuropathology Universitätsklinikum Erlangen Erlangen Germany; ^2^ Consultant for the ILAE Education Council Hamburg Germany; ^3^ Department of Clinical Neurosciences University of Calgary Alberta Canada; ^4^ Department of Clinical Neurophysiology, Danish Epilepsy Centre, Affiliated Partner of the ERN EpiCARE Aarhus University Hospital Aarhus Denmark; ^5^ Department of Clinical Medicine Aarhus University Aarhus Denmark; ^6^ Department of Pediatric Neurology, Red Cross War Memorial Children's Hospital, Neuroscience Institute University of Cape Town Cape Town South Africa; ^7^ Department of Neurology Govind Ballabh Pant Institute of Postgraduate Medical Education and Research‐GIPMER (Delhi University), MAMC New Delhi India; ^8^ Jefferson Comprehensive Epilepsy Center, Department of Neurology Thomas Jefferson University Philadelphia Pennsylvania USA; ^9^ Department of Epileptology (Krankenhaus Mara), Medical School Bielefeld University Bielefeld Germany; ^10^ Paediatric Epilepsy Unit, Neurology Department, Children's Hospital San Juan de Dios, Member of the ERN EpiCARE Universitat de Barcelona Barcelona Spain; ^11^ Department of Neurology Dayanand Medical College and Hospital Ludhiana India

**Keywords:** adaptive learning, antiseizure medication, brain, e‐learning, online learning, seizure

## Abstract

The International League Against Epilepsy (ILAE) Academy is the world's eminent e‐learning campus for epileptology. Its modular teaching content was developed to cover all competencies and learning objectives specified in the ILAE's curriculum for epileptology. The tutorless and self‐paced entry Level 1 program for beginners offers an interactive case‐based e‐learning approach. A blended e‐learning format was developed for the proficiency Level 2 with various learning domains and formats. They comprise a series of interactive, self‐paced and case‐based e‐learning modules covering common, but also rare or complex epilepsy conditions, through state‐of‐the‐art diagnosis and rational treatment decisions. Interactive EEG and MRI readers were integrated to support a tutorless online teaching format. An innovative adaptive e‐learning format was applied for specific learning domains to reflect not only the proficiency level of the learner but also their self‐confidence and perceived and unperceived knowledge of the topic. Our analysis of the completed adaptive e‐learning courses revealed that 21% of the learning objectives had been answered incorrectly despite the learner indicating that they know the answer. This so‐called “unconscious incompetence” should be regarded as a key motivation for building continuing medical education (CME) programs in epileptology. Level 2 utilizes a blended learning approach requiring 200 CME or equivalent ILAE credit points, earned through a mix of online learning with in‐person participation in ILAE schools and congressional teaching activities. Level 3 is the subsequent step in the ILAE's structured learning path towards advanced proficiency and includes skill‐based training in epileptology. Registered learners can apply for training visits in internationally renowned epilepsy centers around the world. Limited financial support is made available for selected applicants, e.g., to support candidates from resource limited settings. Designed to bridge the gap in knowledge and access to continuing education in epileptology, this unique structured learning path is open to all healthcare professionals.

**Key points**

- The ILAE Academy is the world’s eminent e‐learning campus for epileptology. Its modular teaching content was developed to cover all competencies and learning objectives specified in the ILAE’s curriculum for epileptology.
- The Level 1 program is for beginners and offers a tutorless, self‐paced, case‐based e‐learning approach.
- The proficiency Level 2 program utilizes a blended learning approach requiring 200 CME or equivalent ILAE credit points, earned through a mix of online learning with in‐person participation in ILAE schools and congressional teaching activities.
- Level 3 is the subsequent step in the ILAE’s structured learning path towards advanced proficiency and includes skill‐based training in epileptology, e.g., training visits in internationally renowned epilepsy centers around the world.

## 1 INTRODUCTION

While all medical specialties require continuing education and training for skill and competency development, such opportunities are often limited or inaccessible in many parts of the world. Educational activities may occur sporadically and offer neither a structured curriculum nor complete coverage of the subject matter. Moreover, patient care is frequently delivered by non‐specialists or clinicians with diverse responsibilities, including adult neurologists caring for children. This underscores the need for standardized, relevant, and accessible teaching tools, particularly in the complex field of epileptology, where a multidisciplinary approach incorporating neurophysiology, neuroimaging, genetics, pharmacotherapy, and pre‐surgical evaluation is essential.^1^ The broad range of seizure onset from newborns to adults also requires specific knowledge about age‐related conditions and comorbidities. The International League Against Epilepsy (ILAE) has invested substantial effort to establish a globally accessible comprehensive teaching environment for all healthcare professionals who seek continuing medical education in epileptology.^2^, ^3^, ^4^ This article delineates the ILAE Academy's structured learning path and elucidates experiences with its three‐level (1‐2‐3) learning approach.

## 2 THE 1‐2‐3 LEVEL LEARNING APPROACH

**FIGURE 1:**
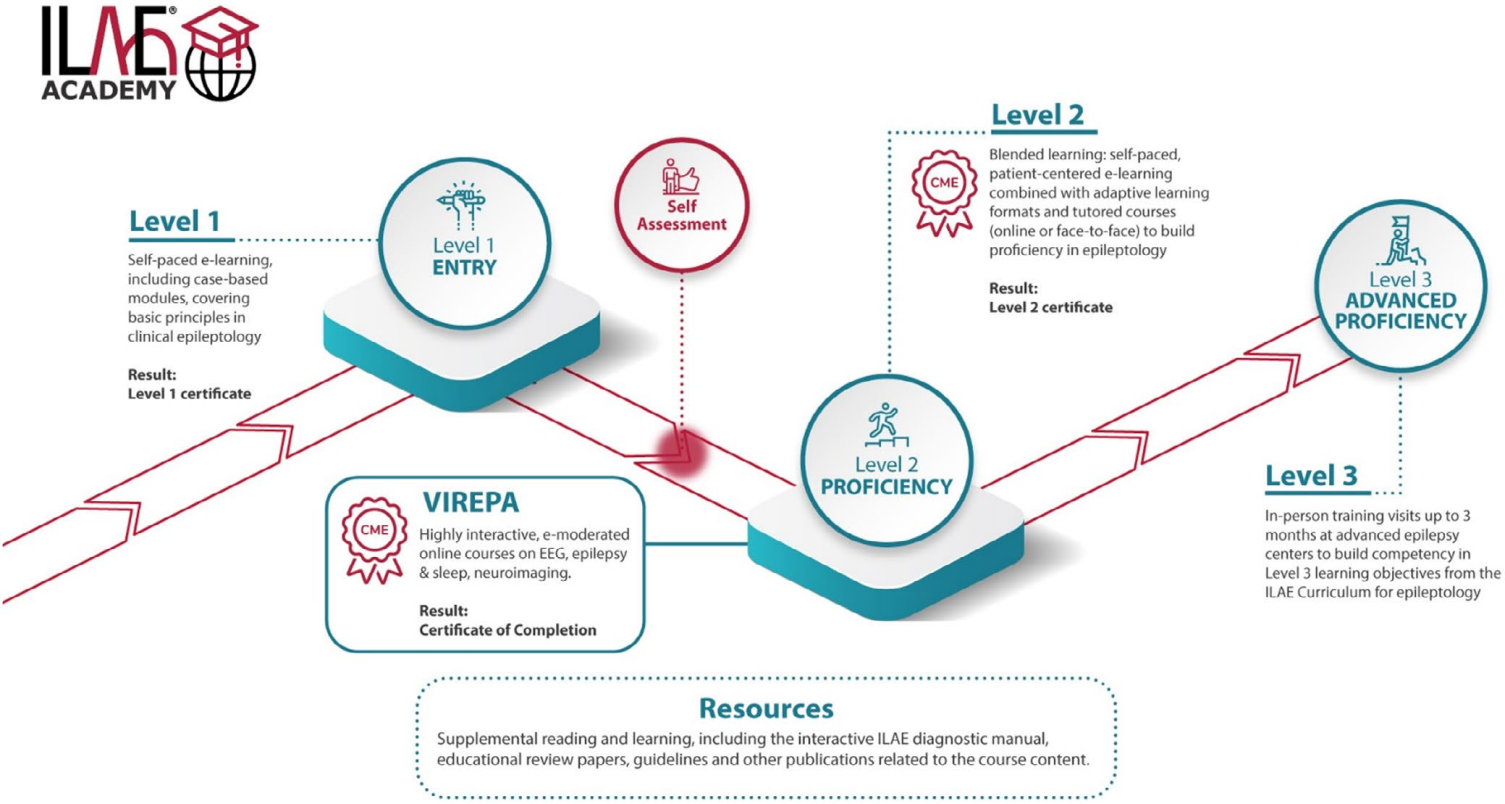
Graphical path of the structured learning program of the ILAE Academy. We thank Jason Ryan (ILAE) for creating this graphic. With permission taken from ilae‐academy.org.

The ILAE educational program is based on a comprehensive curriculum covering 7 domains, 42 competencies, and 124 learning objectives (LO) by consensus among experts from across different regions.^2^ Furthermore, all LOs are grouped into three successive levels starting with the entry Level 1 for beginners, to the proficiency Level 2, and followed by the advanced proficiency Level 3 (Figure 1).

### 2.1 Summary of the entry level 1

**FIGURE 2:**
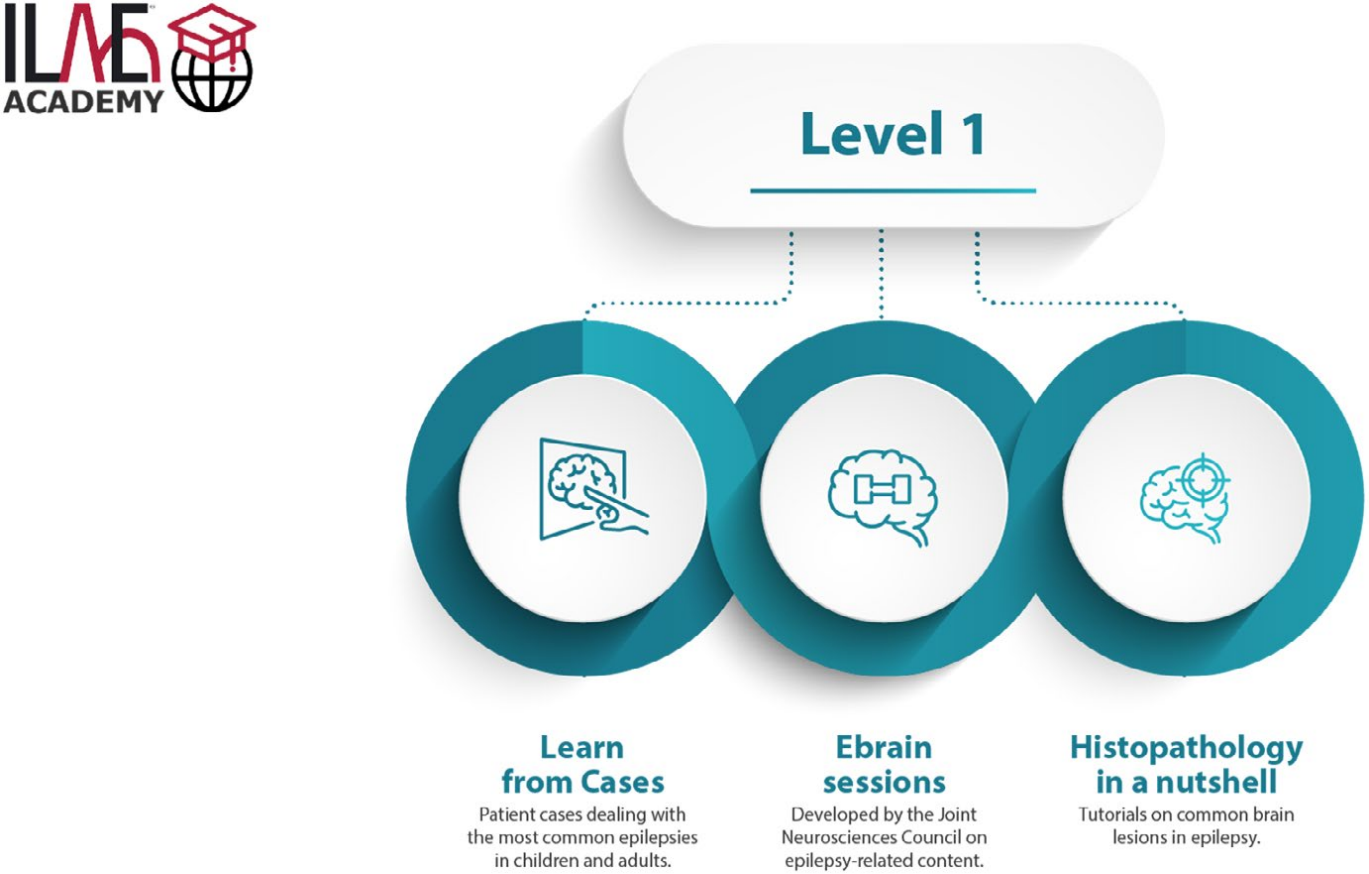
Schematic organization of Level 1 teaching materials of the ILAE Academy. We thank Jason Ryan (ILAE) for creating this graphic. With permission taken from ilae‐academy.org.

The ILAE Academy opened its virtual campus at ilae‐academy.org in July 2020 amidst the COVID pandemic and has successfully trained hundreds of registered users (Table 1 and Figure 2).

**TABLE 1:** ILAE Academy statistics (2020–2024).

ILAE Academy website visitors between 2022 and 2024	57 000
ILAE Academy accounts created	5028
Active users of the ILAE Academy in 2024	1240
ILAE regions/countries (total number)	152

This entry level comprises three main sections: (1) Fifteen highly interactive, self‐paced, case‐based e‐learning courses (“Learn from Cases”) addressing the most common epilepsies in children and adults; (2) a series of short, topic‐specific “ebrain” teaching sessions developed by the Joint Neurosciences Council; and (3) the video course “Histopathology in a nutshell.” All LOs specified in the ILAE curriculum for this level have been addressed, with thousands of course completion certificates issued to date (Table 1). To fully address the comprehensive LOs of the educational curriculum, the e‐learning activities, a series of open access seminars, published in the Epileptic Disorders journal is provided. A one‐year subscription to the ILAE Academy offers access to the entire Level 1 program for a fee of 50–200 USD depending on the learner's country of residence. In addition, learners from resource limited countries can apply for bursaries that allows them to participate in the ILAE Academy teaching program for 25 USD/year. The 15 “Learn from Cases” courses have been translated into Spanish and Chinese, and a French translation will soon be available.

### 2.2 Summary of the proficiency level 2

**FIGURE 3:**
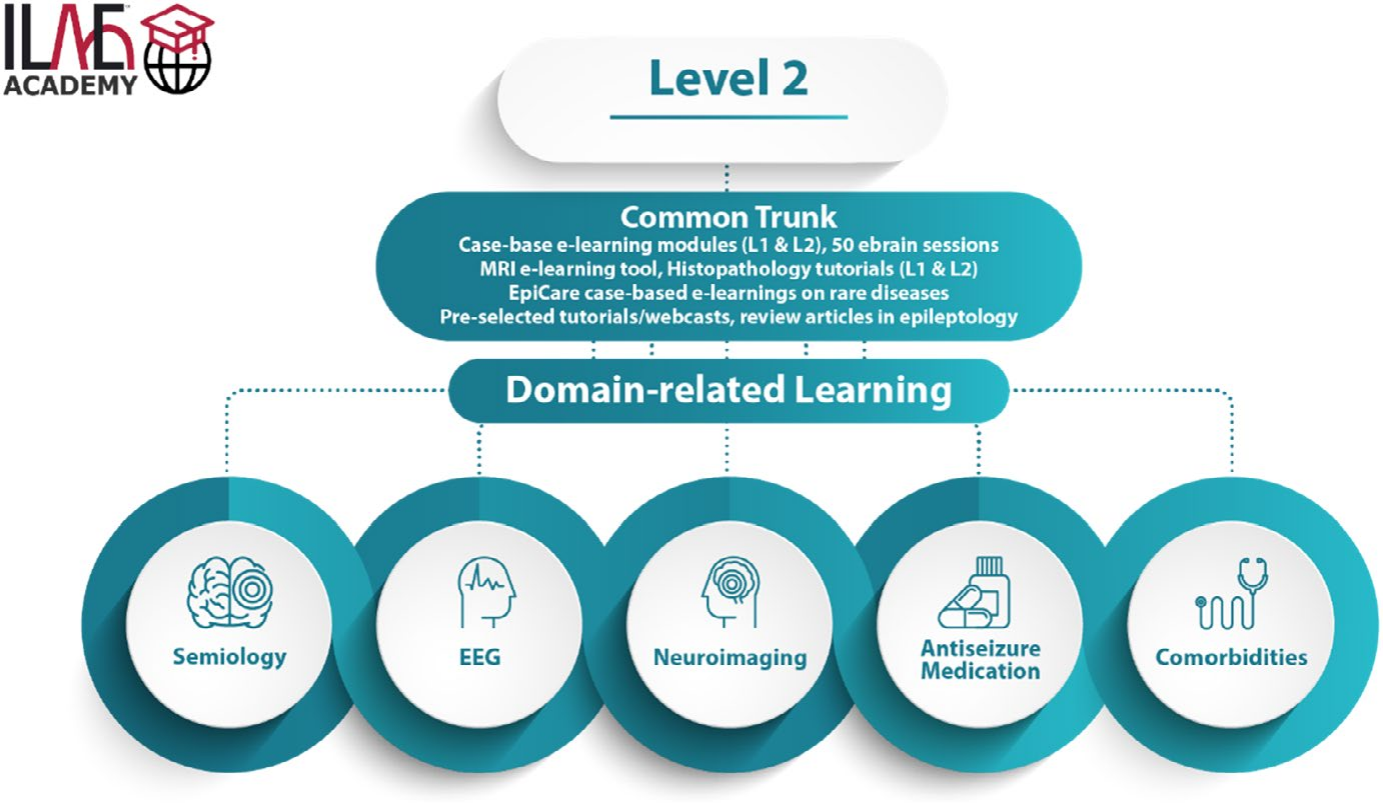
Schematic organization of Level 2 teaching materials of the ILAE Academy. We thank Jason Ryan (ILAE) for creating this graphic. With permission taken from ilae‐academy.org.

The ILAE Academy learning platform was updated in 2022. This involved important enhancements to the functionality of ILAE's teaching portfolio required for Level 2 (“proficiency level”), as well as access to additional teaching programs of the ILAE.^4^ As with Level 1, the Level 2 program followed the principles of adult education,^5^ including practical relevance, goal‐orientation, and outcome‐based learning. As outlined in the published ILAE curriculum roadmap,^2^ the proficiency Level 2 has substantially deeper and broader coverage of knowledge and skills in epileptology than Level 1 (Figure 3). Level 2 learning activities target advanced competency domains of the educational curriculum, including semiology, EEG, medical treatment and comorbidities in patients with seizures and epilepsy. These activities are aimed at enhancing learners' ability to make diagnostic decisions, counsel patients, choose appropriate antiseizure medications (ASMs), and manage psychiatric and somatic comorbidities. This is supported by a comprehensive portfolio of case‐oriented e‐learning modules covering both the main epilepsy types and syndromes, and less common epileptic disorders (Table 2). An interactive online EEG and MRI reader was integrated into the e‐learning modules to enable training in use and interpretation of diagnostic tools based on realistic case scenarios and exercises. The challenging subject of ictal semiology is a cornerstone for the correct classification of seizures and epilepsies. In addition to the semiological aspects embedded in the patient cases, this area is extensively addressed through specific modules with illustrative video presentations, discussions, exercises, and a detailed semiology glossary supported by illustrative video examples.^6^ Please also see “GET A GLIMPSE” on the landing page of the ILAE Academy website for further illustration (www.ilae‐academy.org). This comprehensive, state‐of‐the‐art online teaching program is unprecedented and has received overwhelmingly positive feedback and testimonials from Level 2 learners. Each participant was asked to submit their feedback after finishing a course module using a questionnaire template based on CME relevant parameters for the final learning activity evaluation. This questionnaire also included a free text option to comment on their personal experience with this e‐learning activity. A total of 196 responses specifically addressed the format and content of the course and were used for our further evaluation. From these responses, 47% were enthusiastically positive and 6% were negative. The remaining 47% of free text responses addressed hints regarding errors, phrasing, technical (IT) performance or suggestions for further e‐learning activities and content development.

**TABLE 2:** Course portfolio of Level 2.

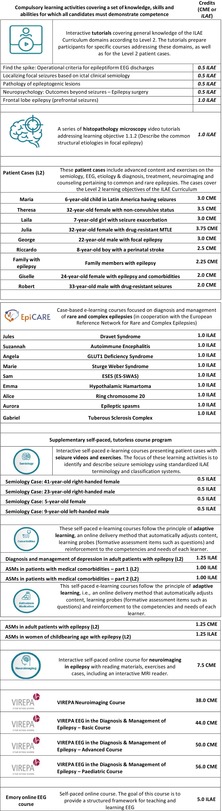

*Note*: CME credit points were obtained in cooperation with Albert Einstein College of Medicine‐Montefiore Medical Center, New York/ NY, USA, as jointly accredited by the Accreditation Council for continuing Medical Education (ACCME) as *AMA PRA Category 1 Credits™*. ILAE credit points were granted using similar guidelines, including the presumed learning hours to accomplish the course.

Abbreviations: ASMs, antiseizure medications.

The Level 2 e‐learning program encompasses three sections: (1) A Common Trunk consisting of self‐paced, tutorless online modules which are compulsory for all registered learners (Table 2). (2) Additional online and tutorless teaching offerings which address key Level 2 competencies, e.g., semiology, ASMs, comorbidities, neuroimaging, and EEG as optional subjects (Table 2). (3) An online program that blends in tutor‐guided live tutorials and web‐based teaching courses (e.g., VIREPA courses, the Baltic ILAE school, neuroimaging ILAE school), and advanced face‐to‐face workshops (e.g., ILAE schools for neuropsychology, neuropathology, EEG in neonates, advanced EEG, and neuroimaging). The ILAE website provides further information including course schedules or special teaching courses offered at international and regional ILAE congresses (www.ilae.org). Learners who complete the Level 2 program obtain an ILAE certificate after they accrue 200 CME credit points (equivalent to ILAE credit points). Of these, 100 credit points must be obtained from the Level 2 online modules, which include all offerings from the common trunk as well as additional teaching courses selected individually by the learner (Table 2). The remaining 100 credit points must be obtained from tutored ILAE courses which are offered online (e.g., VIREPA courses on EEG or MRI), in person (e.g., ILAE schools, workshops), and face‐to‐face training courses at ILAE congresses (see ILAE website for all available courses and updated time schedules). More information on how to use tutorials, current ILAE fee structures, and available bursaries can be found in the ILAE Academy website at www.ilae‐academy.org.

### 2.3 Five adaptive e‐learning modules are offered in Level 2

We introduced the adaptive e‐learning format^7^ to the Level 2 educational portfolio to focus on specific knowledge‐based LOs and domains, e.g., antiseizure medications and comorbidities (Table 3). The concept of adaptive learning is an educational approach that personalizes the learning experience by adjusting the pace, content, and instructional paths based on the learner's individual needs, skills, and progress. Through the use of data analytics, Artificial Intelligence (AI) algorithms and real‐time feedback, adaptive learning systems can identify the learner's strengths and weaknesses and respond individually by providing customized resources or exercises.^8^, ^9^ This approach aims to optimize the learner's motivation, improve efficiency and personalized knowledge transfer, as well as saving time through retention of confidently known learning material.^9^, ^10^, ^11^ Adaptive learning can be applied both in traditional classrooms and in digital learning environments. The adaptive e‐learning software used by the ILAE Academy was developed using state‐of‐the‐art expertise provided by Area9 Lyceum. Area9 has implemented Bloom's approach and scaled it with an AI‐driven adaptive learning algorithm.^12^ This uses granular learning objectives, cognitive mapping and other factors such as detailed measures of proficiency, confidence, time, self‐perception in their tendencies to accurately measure each learner's strengths and weaknesses. This format adapts the presentation of questions (probes) and teaching material according to the learner's self‐assessment as being confident or not confident in making the correct decision when answering a topic‐specific probe.^11^, ^13^ Based on these results, the algorithm determines which content or learning objective needs more emphasis, where remediation is needed and where no additional learning time should be spent on superfluous material. These measurements are performed directly whilst progressing through the teaching module and adapted to each individual learner's needs. The technology repeatedly challenges the learner and generates reporting data that continuously document learning progress (https://area9lyceum.com/adaptive‐learning/#; aithority.com. 29 January 2019). Area9 assesses this approach through the continual tracking of metacognition, which is defined as the awareness of one's own thinking and learning (https://en.wikipedia.org/wiki/Metacognition, accessed 16 December 2024). Analyses of these data provide insights into the learner's understanding of the topic and their level of confidence in their knowledge.^14^, ^15^ Thus, metacognition plays a crucial role in Area9's adaptive approach and the algorithm helps learners to become more proficient, while uncovering their hidden misconceptions. To capture the learner's metacognition during the course progress, they are asked to check their self‐confidence after answering each probe. The four self‐assessment options offered for each probe are:

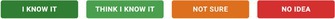

**TABLE 3:** Initial metacognition analysis of ILAE's adaptive e‐learning modules.

Course module	Conscious competence	Unconscious competence	Conscious incompetence	Unconscious incompetence	Total
Depression	60%	15%	5%	21%	100%
ASM PWE	55%	7%	6%	33%	100%
ASM Women	82%	1%	2%	15%	100%
ASM Comorb 1	72%	3%	6%	20%	100%
ASM Comorb 2	82%	1%	1%	16%	100%

*Note*: Numbers are rounded to the nearest whole number.

Metacognition is categorized according to these competence criteria into (1) conscious competence, i.e., learners answer correctly and state that they have the knowledge, (2) unconscious competence, i.e., learners answer correctly and state that they are not sure or do not have the knowledge, (3) conscious incompetence, i.e., learners answer incorrectly and state that they are not sure or do not have the knowledge, and (4) unconscious incompetence, i.e., learners answer incorrectly and state that they have the knowledge. The required final score for successful completion of these courses was set at 100%, thus the adaptive model supports learners to master the missing knowledge by repeating all of the missed learning objectives during their learning experience until they reach the level of conscious competence.

In our courses, there are 17 probes for depression, 18 for ASMs in persons with epilepsy (PWE), 23 for ASMs in women, and 20 for ASMs and comorbidities, which are divided into two parts. The rationale for choosing these five specific topics from the ILAE curriculum for the adaptive e‐learning courses in the ILAE Level 2 program (Tables 2 and 3) is as follows: (1) Depression is a critically important psychiatric comorbidity in epilepsy; (2) Use of ASMs in PWE is a core competence in clinical epileptology; (3) ASM use in women with epilepsy of childbearing potential has significant implications for family planning and offspring health; (4) ASMs in PWE and somatic comorbidities have great relevance when treating chronically ill individuals, and is divided into two parts (ASM Comorb. 1 and 2). The selected learning objectives from the ILAE curriculum provided a suitable framework for adaptive learning, allowing assessment of learners' initial competence and subsequent adaptation of content and assessments based on their progress. The transfer of knowledge is tested predominantly using scenario‐based questions. In addition, the learner is guided by a digital coach and receives repetition and additional information to reinforce the learning effect to ultimately achieve 100% evidence of mastery. Evaluation of the learning analytics revealed interesting insights into learners' competency development and learning progress. From the outset, the focus is on learners' self‐assessment regarding proficiency, their learning progress and metacognition data. Upon entering a course and prior to starting the probes, learners are asked to estimate their self‐perceived level of proficiency (novice, advanced beginner, competent, proficient, or expert). The metacognition reports presented here (Table 3) assessed the relationship between learners' answer accuracy on the probes and their self‐reported confidence in those answers. Only learners who had successfully completed a course from November 2022 (first course implemented) until July 2024 are included, i.e., those that achieved levels of 100% conscious competence.

Evaluation of the initial metacognition, defined as the first interaction with each learning objective (probe) for all learners of this course, revealed that on average, 21% of learners through all proficiency cohorts and all courses were evaluated as unconsciously incompetent, i.e., answering incorrectly while stating they know the answer (Table 3). The topic of answers is unraveling as they cover core competencies in clinical epileptology, e.g., management of depression as one major psychiatric comorbidity as well as ASM‐induced depression (Table 4). Learner feedback highlighted the need for further training in the use of common rating scales, such as the Neurological Disorders Depression Inventory for Epilepsy (NDDI‐E), which is presented in this module and is widely accessible, having been translated and validated in many languages.^16^ Understanding the mechanisms of ASM interactions constitutes a key competency in clinical care; however, this domain revealed the highest degree of unconscious incompetence, highlighting an urgent need for further training and education. Similarly, the management of ASMs in women of childbearing age necessitates a careful assessment of the risk–benefit balance between achieving seizure control and the risk of neurodevelopmental disorders (Table 4). The use of ASMs in the aging population of PWE is complicated by the increase of somatic comorbidities in this population. Hepatic impairment and diabetes mellitus present particular management challenges, especially when additional conditions such as obesity, migraine, or even cancer are present (Table 4). The adaptive e‐learning approach serves to elucidate topics requiring further guidance to the ILAE Education Council in the development of targeted e‐learning offerings.

**TABLE 4:** Selection of the most difficult LOs per course based on the metacognition analysis.

Course	Learning objective	Unconsciously incompetent
Depression	Recognize the management of suicidality in patients	50%
Depression	List the treatment steps of ASM‐induced depression	26%
Depression	Match the treatment to the forms of interictal depression	25%
ASM PWE	Identify the mechanisms of drug interactions with ASMs	51%
ASM PWE	Identify the variables that affect a specific ASM's appropriateness for a PWE	46%
ASM PWE	Recognize the indications for the use of valproate in pre‐menopausal women	45%
ASM PWE	Identify the most common ASMs for the 2nd and 3rd line treatment in focal epilepsy	44%
ASM Women	Identify when should valproate be offered as initial treatment if valproate is considered as the most appropriate therapy for a particular type of epilepsy	53%
ASM Women	Identify the ASMs with low risk of neurodevelopmental disorders in children when used by pregnant women	30%
ASM Women	Identify the correct consideration regarding delivery time for pregnant women with epilepsy without underlying obstetric risk factors	28%
ASM Comorbidities 1	List the preferred ASMs in patients with epilepsy and severe hepatic impairment	30%
ASM Comorbidities 1	List the preferred ASMs in patients with epilepsy and diabetes mellitus	29%
ASM Comorbidities 1	Differentiate between suitable and non‐suitable ASMs for patients with renal impairment	28%
ASM Comorbidities 2	List the ASMs to avoid in patients with epilepsy and obesity	39.3%
ASM Comorbidities 2	List the preferred ASMs in patients with epilepsy and migraine headaches	35.5%
ASM Comorbidities 2	Identify pathologies that are the possible etiology of seizures in patients with cancer	25%

*Note*: See text for abbreviations.

Ultimately, all these course participants achieved the 100% goal of conscious competence with regard to the learning objectives of the respective course through the adaptive learning intervention facilitated during the course progress. Individual reports on personal initial, current and improved metacognition and related learning objectives can be retrieved by the learner to gain deeper insight into their competencies achieved. In addition, the learner can access the module for some time after completing the course and is then automatically challenged with their most difficult learning objectives in order to repeat them and reinforce the knowledge.

### 2.4 Level 2 program accomplishment achieved

The completion of Level 2 marks a significant milestone for the ILAE Academy in its pursuit of becoming the world's premier learning objective (LO)‐based educational portfolio in clinical epileptology. The overwhelmingly positive feedback received from the learners, e.g., 47% of the 196 received feedback commentaries were ranked as enthusiastically positive compared to 6% being negative and 47% regarded errors, phrasing, technical (IT) performance or had other content, attests to the ILAE Education Council's dedicated efforts in planning, programming, and financial resources required to develop 21st century state‐of‐the‐art teaching material. These digital course modules can also be used in other teaching programs, either online or face‐to‐face, and can be shared with future ILAE‐sponsored educational programs upon request to the Education Council. The program's division into three levels—from entry level (L1) to proficiency (L2) and culminating in advanced proficiency (L3)—reflects a targeted learning design. This design utilizes online‐only materials in L1, a blended tutorless/tutored format in L2, and a skill‐based training program in L3, which allows participants to observe and train in advanced epilepsy centers around the world (see below).

### 2.5 The launch of level 3 of the ILAE academy (December 2024)

The structured, progressive course portfolio enables ILAE Academy trainees to advance their careers in clinical epileptology from foundational knowledge to advanced proficiency. Consequently, Level 3 must comprehensively address all 124 learning objectives (LOs) specified in the ILAE curriculum to ensure the highest standards of patient care globally. A Level 3 teaching portfolio is in theory limitless in scope and potential to address the needs of individual learners. After considering various program options, cost–benefit analyses, and timelines, a Task Force of the ILAE Education Council identified five key areas requiring specialized training to achieve advanced proficiency: (1) Epilepsy Surgery, (2) Neonatal and Pediatric epileptology, (3) Clinical Trials, (4) Neurogenetics, (5) Basic and clinical research. The Task Force also recognized the necessity of acquiring and consolidating practical competencies within a clinical setting to fully realize the objectives of the ILAE training cycle and to complement the predominantly online instructional approach. Accordingly, the ILAE Executive Committee authorized financial support for Level 2 certificate recipients to undertake training in diagnostic and therapeutic skills at advanced epilepsy centers internationally, with preference given to placements within their respective home regions. This aligns with Goal 2 of the Mission of the ILAE, which is to support health professionals worldwide to enhance their knowledge and skills in the prevention, diagnosis, treatment, and care of epilepsy. Applications for the Level 3 training visits must be submitted through the ILAE website using the provided template documents (https://www.ilae.org/education/ilae‐academy) and the application forms available at https://www.surveymonkey.com/r/L3TrainingVisit. Applicants should adhere to the following procedures:

1. Individuals applying for training visits should specify curricular competencies and learning objectives based on the ILAE Epileptology Curriculum that they hope to address during the training visit.
2. The Host Institution/Department in turn should clarify if these learning objectives would be suitably met at the Institution/Department in the given period of time.
3. Under all circumstances, trainees are expected to return to their home institution/country at the end of the training visit in order to practice the learnings acquired.
4. A structured evaluation and feedback from both the trainee and the host department/institution should be provided to the ILAE Regional Board at the end of the training visit. Both parties might use a template form provided by the ILAE for this purpose.
5. The ILAE might seek to assess the outcomes of the training visit from the trainee in the long term (2–5 years later).

Applications are accepted on a rolling basis until funding is exhausted. Applicants who have obtained an ILAE Level 2 certificate will receive preferential consideration when applying for ILAE bursaries to support further educational opportunities. A comprehensive list of educational offerings is available on the ILAE website. This will not only fulfill the advanced proficiency level requirements of Level 3 but also enhance the competitiveness of candidates for review committees. For example, ILAE Academy Level 2 certificate holders may receive preferential consideration when applying for the *Visiting Scholarship Program* offered by the ILAE Career Development Commission.

The development of new online teaching materials for Level 3 is currently under consideration by the ILAE Executive Commission and Education Council, pending the availability of necessary human and financial resources. Any decisions regarding Level 3 online learning will be announced through ILAE's official channels, including social media, newsletters, and the ILAE website.

### 2.6 Further learning activities on the ILAE academy

As a leading global platform for epilepsy education, the ILAE Academy continuously evolves its offerings beyond the core curriculum. This dynamic platform now encompasses a wide range of specialties, including primary care, nursing, radiology, and more, ensuring that learners have access to the most up‐to‐date knowledge and best practices in the field of epileptology. A comprehensive online course for primary care in epileptology, based on the ILAE's new curriculum for primary care, will soon be available.^17^, ^18^ The ILAE Academy has successfully integrated an online program previously developed by ALADE (Academia Latino Americana de Epilepsia), the Latin American Regional Board of the ILAE. Stay informed about the latest educational opportunities in epileptology by subscribing to the ILAE newsletter and following the ILAE social media channels.

## CONFLICT OF INTEREST STATEMENT

IB, EB, AAP, AA have nothing to disclose. CB has received support from and/or has served as a paid consultant for Angelini, Jazz Pharmaceuticals, Johnson & Johnson, Marinus, and Xenon Pharma. JMW is an associate editor for Epilepsia. SW has received unrestricted educational grants on behalf of his institution from UCB Pharma, Sunovion, Eisai, Paladin Labs, and Jazz Pharma, and has received honoraria or served on advisory boards for Paladin Labs, Jazz Pharma, and Torrent Labs.

## DISCLAIMER

This report was written by experts selected by the International League Against Epilepsy (ILAE) and was approved for publication by the ILAE. Opinions expressed by the authors, however, do not necessarily represent the policy or position of the ILAE.

## Data Availability

Data sharing is not applicable to this article as no new data were created or analyzed in this study.

## APPENDIX A

Ad hoc faculty members of the ILAE Academy Level 1 and Level 2 online programs (*in alphabetical order*).

Raidah Al Baradie, MD, King Fahad Specialist Hospital, Saudi Arabia; Carmen Barba, MD, PhD, Meyer Children's Hospital IRCCS*, University of Florence, Florence, Italy; Jocelyn F. Bautista, MD Cleveland Clinic Lerner College of Medicine, USA; Sallie Baxendale, PhD, UCL, Queen Square Institute of Neurology and National Hospital for Neurology & Neurosurgery, London, UK; Elena Belousova, MD, PhD Russian National Research Medical University n.a. N.I.Pirogov, Russia; Isabella Brambilla, ePAG ERN EpiCare Coordinator, Dravet Italia; Jorge G. Burneo, MD, MSPH, Western University & London Health Sciences Center, ON, Canada; Leonor Cabral‐Lim, MD, Philippine General Hospital, University of the Philippines Manila, Philippines; Jaime Carrizosa, MD, Mapeo Genético Research Group, University of Antioquia, Medellín, Colombia; Jaume Campistol, MD, PhD, Hospital Universitary Sant Joan de Deu de Barcelona*, Spain; Hannah Cock, MBBS MD, City St George's University of London and St George's University Hospitals NHS Trust, London, UK; Marion Comajuan, MD, Hopital Femme Mère Enfant*, Lyon, France; Helen Cross, MB ChB Phd FRCPCH FRCP, UCL Great Ormond Street Institute of Child Health*, London, UK; Emilija Cvetkovska, MD, PhD, St Cyril and Methodius University in Skopje, North Macedonia; Valentina De Giorgis, MD, PhD, Fondazione Mondino, Istituto Neurologico Nazionale*, IRCCS, Pavia, Italy; Luca De Palma, MD, PhD, Bambino Gesu Children Hospital*, Rome, Italy; Kirsten A. Donald, MD, PhD, University of Cape Town, South Africa; Georg Dorfmüller, MD, Rothschild Foundation Hospital*, Paris, France; Guadalupe Fernandez Baca Vaca, MD, Case Western Reserve University, University Hospitals Cleveland Medical Center, USA; Jessica Fesler, MD, Cleveland Clinic, Ohio, USA; Irene García Morales, MD, PhD, Hospital Clínico San Carlos, Spain; Christina Giavasi, MD, MSc, Southmead Hospital, North Bristol NHS Trust, Bristol, UK; Maria Gogou, MD, PhD, MSc, Evelina Children's Hospital & King's College Hospital, London, UK; Patricia Gómez Iglesias MD, Hospital Universitario de Torrejón, Spain; Samson Gwer, MBChB FRCPCH PhD, Kenyatta University, Kenya; Till Hartlieb, MD, Center for Pediatric Neurology, Neuro‐Rehabilitation and Epileptology, Schoen Klinik Vogtareuth, Germany, and Research Institute Rehabilitation, Transition, Palliation, PMU Salzburg, Austria; Martin Holtkamp, MD, Charité ‐ Universitätsmedizin Berlin, Germany; Floor E. Jansen, MD, PhD, Brain Center University Medical Center*, Utrecht, the Netherlands; Bosanka Jocic‐Jakubi, MD, PhD, Dar Al Shifa Hospital, Kuwait and Pediatric Neurology Clinic “Neuro Kid Bossa”, Nis, Serbia; Philippe Kahane, MD, PhD, University Hospital*, Grenoble, France; Ioannis Karakis, MD, PhD, MSc, Emory University School of Medicine and University of Crete School of Medicine, Crete, Greece; Takafumi Kubota, MD, Tohoku University Hospital, Japan; Joshua Laing, MBBS FRACP PhD, Monash University, Australia; Tatjana Liakina, MD, Sahlgrenska University Hospital*, Gothenburg, Sweden; Aileen McGonigal, MD, PhD, MRCP, Mater Hospital, Brisbane and University of Queensland, Australia; Rosa Michaelis, MD, University Witten/Herdecke; Ruhr epileptology, Knappschaft Kliniken University Hospital Bochum, Germany; Ioana Mindruta, MD, PhD, University Emergency Hospital of Bucharest*, School of Medicine, University Carol Davila Bucharest, Romania; Andrew Neal, MBBS PhD, Monash University, Australia; Maureen Njoroge, MD, University of Nairobi, Thika Level 5 Hospital, Kenya; Irina Oane, MD, University Emergency Hospital Bucharest, Romania; Terence O'Brien, MD, FRACP, Monash University, Australia; Finbar O'Callaghan, MD, PhD, MA, MB ChB, MSc, Institute of Child Health, University College London, UK; Manuela Ochoa Urrea, MD, University of Texas Health Science Center at Houston, USA; Nicholas Odero, MD, Nyamira County Referral Hospital, Kenya; Sebastián Ortiz, MD, MSc, Department of Epilepsy Genetics and Personalized Treatment, Danish Epilepsy Centre, Filadelfia*, Dianalund, Denmark, Filadelfia, Denmark, Department of Regional Health Research, University of Southern Denmark, Odense, Denmark, Departamento de Neurología Pediátrica, Instituto Roosevelt, Bogotá, Colombia; Karine Ostrowsky‐Coste, MD, PhD, Department of Pediatric Epileptology and Neurophysiology, Hospices Civils de Lyon*, France; Maria T. Papadopoulou, MD, Department of Pediatric Epileptology and Neurophysiology Hospices Civils de Lyon*, France; Tiziana Pisano, MD, Meyer Children's Hospital IRCCS*, Florence, Italy; Anna Rada, MD, Department of Epileptology, Krankenhaus Mara, Bethel Epilepsy Center, Medical School OWL, Bielefeld University, Bielefeld, Germany; Stefan Rampp, MD; Department of Neurosurgery and Department of Neuroradiology, University Hospital Erlangen*, Germany; Markus Reuber, MD PhD, Academic Neurology Unit, University of Sheffield, Sheffield, UK; Sylvain Rheims, MD, PhD, University of Lyon, Department of Functional Neurology and Epilepsy*, Lyon, France; Andrea O. Rossetti, MD, University of Lausanne, Switzerland; Guido Rubboli, MD, PhD, Danish Epilepsy Center in Dianalund* and University of Copenhagen, Denmark; Victoria San Antonio‐Arce, MD, PhD, Epilepsy Center, University Medical Center of Freiburg*, Germany; Jan‐Christoph Schoene‐Bake, MD, PhD, Medizinische Hochschule Hannover, Germany; Artem Sharkov, MD, Veltischev Research and Clinical Institute for Pediatrics and Pediatric Surgery of the Pirogov Russian National Research Medical University, Moscow, Russia; Hugh D. Simpson, MBBS, PhD, Department of Neurology, Alfred Health, and Department of Neuroscience, Monash University, Melbourne VIC, Australia; Mary Lou Smith, PhD, University of Toronto and The Hospital for Sick Children, Toronto, Canada; Ana Suller Marti, MD, PhD, Western University, ON, Canada; Rainer Surges, MD, MHBA, Department of Epileptology, University Hospital Bonn*, Germany; Roland Thijs, MD PhD FRCP, Stichting Epilepsie Instellingen Nederland (SEIN), The Netherlands; Joseph Toulouse, MD, Department of Pediatric Epileptology and Neurophysiology*, University Hospital of Lyon, France; Bart van den Munckhof, MD, PhD, Erasmus Medical Centre Rotterdam, The Netherlands; Bernd J. Vorderwülbecke, MD, Charité ‐ Universitätsmedizin Berlin, Germany; Alistair Wardrope, MRCP, Sheffield Teaching Hospitals NHS Foundation Trust, Sheffield & University of Sheffield, UK; Peter Wolf, MD, PhD, Danish Epilepsy Centre Filadelfia*, Denmark; Elza Márcia Yacubian, MD, PhD, Universidade Federal de São Paulo, Brazil.

*Member or Supporting partner of the European Reference Network for Rare and Complex Epilepsies, ERN EpiCARE.
